# Comparison of porcelain veneer fracture in implant‐supported fixed full‐arch prostheses with a framework of either titanium, cobalt–chromium, or zirconia: An in vitro study

**DOI:** 10.1002/cre2.558

**Published:** 2022-03-21

**Authors:** Märta Vahnström, Petra H. Johansson, Per Svanborg, Victoria F. Stenport

**Affiliations:** ^1^ The Brånemark Clinic, Public Health Service Region of Västra Götaland Gothenburg Sweden; ^2^ Department of Prostodontics/Dental Materials Science, Institute of Odontology, Sahlgrenska Academy Gothenburg University Gothenburg Sweden

**Keywords:** fracture strength, implant‐supported prostheses, porcelain veneer

## Abstract

**Objectives:**

The aim of this study was to compare porcelain veneer strength on screw‐retained implant‐supported fixed full‐arch prostheses with a framework of either milled titanium (Ti), cobalt–chromium (CoCr), and yttria‐stabilized zirconia (Y‐TZP) in an in vitro loading model.

**Materials and Methods:**

Fifteen screw‐retained maxillary implant‐supported full‐arch prostheses (FDP), five each of Ti, CoCr, and Y‐TZP frameworks with porcelain veneers were included. All FDPs were subjected to thermocycling before loading until fracture of the veneer. The load was applied at the distal fossa of the occlusal area of the pontic replacing 24. Fracture loads were analyzed, and the fracture quality was assessed. Statistical analysis on the fracture load was performed using Kruskal–Wallis test. The statistical significance was set at *p* < .05.

**Results:**

There was no statistical significance found between the groups regarding fracture load. The highest and lowest load was seen within the CoCr FDP, varying between 340 and 1484 N. Different types of fracture appearances were seen. The Y‐TZP FDPs had a higher number of fractures locally in the loaded area while CoCr and Ti more often showed cracks in the anterior region, at a distance from the loaded area.

**Conclusions:**

Within the limitations of this study, the conclusion was that framework material may affect the fracture behavior of maxillary full‐arch bridges; however, there were no differences in veneer fracture strength when frameworks of Ti, CoCr, or Y‐TZP were compared.

## 1 INTRODUCTION

In 1965 Per‐Ingvar Brånemark treated his first edentulous patient with an implant‐supported fixed dental prosthesis (FDP), since then the treatment has been widely used in situations with missing teeth and is considered a predicable treatment (Astrand et al., 2008; Brägger et al., 2001; Chrcanovic et al., 2020; Karlsson et al., 2018; Papaspyridakos et al., 2012; Pjetursson et al., 2012; Riemann et al., 2019; Romeo & Storelli, 2012; Sailer et al., 2018).

High survival rates have been reported for implant‐supported prosthetics but there is a high frequency of complications, both biological and technical. Technical complications that have been described in the literature are: implant fracture, screw fractures, and screw loosening, among others. The most common technical complication reported is the fracture of the veneering material (Pieralli et al., 2018; Pjetursson et al., 2012; Romeo & Storelli, 2012).

Implant‐supported FDPs have higher rates of porcelain veneer fractures compared to tooth‐supported FDPs (Brägger et al., 2001). It has been pointed out that bruxism causes an increased risk of porcelain fractures in implant‐supported prostheses (Brägger et al., 2001; Chrcanovic et al., 2020; Kinsel & Lin, 2009). One reason for the higher risk of veneer fracture in implant‐supported FDPs could be that implant fixtures do not have periodontal ligaments and therefore have less proprioception and resiliency, resulting in higher chewing loads (Higaki et al., 2014; Schulte, 1995). Hence, a higher load in implant restorations is a probable cause for more frequent veneer fractures. Although, veneer fractures may occur due to insufficient support of the porcelain veneer. The thickness of the veneer may as well effect the strength of the veneer. Generally, porcelain should not have a higher dimension than 2.0 mm on either Y‐TZP or metallic frameworks. The optimal thickness is considered 1 mm (Bakitian et al., 2017).

A veneer fracture can be either adhesive, in the interface between the veneering porcelain and the framework material, or cohesive, within the veneering material itself. Fracture of the veneer is often referred to as chipping (Karlsson et al., 2018; Papaspyridakos et al., 2012; Papia & Larsson, 2018). Different materials are being used for the framework. In earlier years, gold alloys with acrylic teeth were the gold standard but today titanium (Ti), cobalt–chromium (CoCr), and yttria‐stabilized zirconia (Y‐TZP) with porcelain veneers are more frequently used. A systematic review by Pjetursson et al. (2012) showed that veneer fracture complication rate was 20.2% in partial FDPs with gold‐acrylic FDPs and 7.8% in FDPs veneered with porcelain (metal and zirconia frameworks) in a 5‐year prospective study (Pjetursson et al., 2012), while other researchers found even higher numbers of chipping, the cumulative rate at 33.3% at 5 years and 66.6% at 10 years when studying full‐arch FDPs only, mainly metal‐resin FDPs (Papaspyridakos et al., 2012). Other studies have confirmed the fracture of the veneering material as the most common technical complication with a range between 14% and 36% (Papia & Larsson, 2018; Riemann et al., 2019). With the development of new materials more and more porcelain veneered frameworks are in use. In a systematic review by Sailer et al. (2018), the estimated 5‐year rate of porcelain fractures of metal‐porcelain FDPs was 11.6% while the rate for zirconia‐porcelain FDPs was significantly higher, 50%, of the zirconia‐porcelain indicating the importance of the framework material.

Chipping of the veneer is most often a minor complication and may often be solved by polishing the fractured area; however, in some cases, it is not possible, and the fracture of the veneer can lead to the replacement of the prosthesis (Karlsson et al., 2018; Koenig et al., 2013). Furthermore, patients who have suffered from a fracture of the veneer are significantly less satisfied with their treatment than those without complications (Koenig et al., 2013). This combined with the fact that fracture of the veneering material is the most common complication emphasizes the importance to the consider choice of material for the prosthesis in order to avoid veneer fractures. As yet, to our knowledge, no randomized controlled clinical studies regarding the choice of material to minimize technical complications has been published. No randomized controlled clinical trials, comparing veneer fractures of metal porcelain and zirconia‐porcelain full‐arch implant‐supported FDPs were available at the time this study was conducted. Therefore, investigations of various framework materials regarding veneer fractures are needed.

The aim of this study was to compare porcelain veneer strength on screw‐retained implant‐supported fixed full‐arch prostheses with a framework of either milled Ti, CoCr, and Y‐TZP in an in vitro loading model with a null hypothesis that there is no difference between the framework materials with respect to porcelain strength.

## 2 MATERIALS AND METHODS

### 2.1 Design and specimens

**Figure 1:**
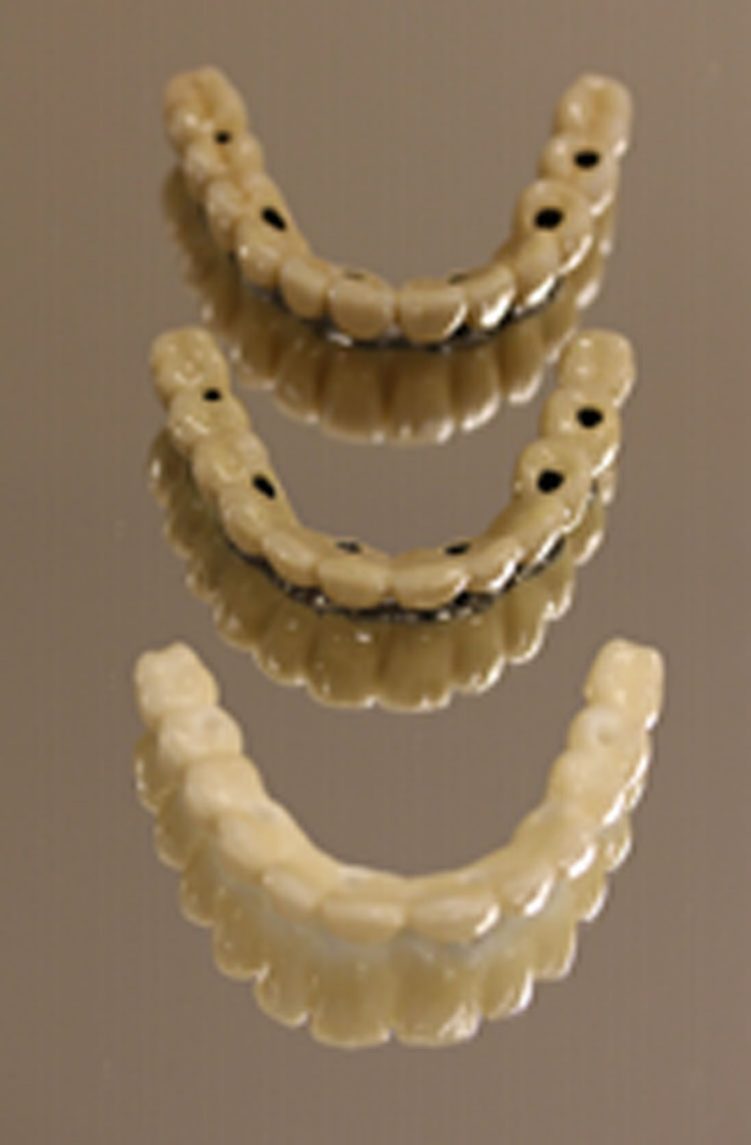
The three different types of frameworks. From top to bottom, CoCr, Ti, Y‐TZP. CoCr, cobalt–chromium; Ti, titanium; Y‐TZP, yttria‐stabilized zirconia

In total, 15 screw‐retained maxillary implant‐supported full‐arch prostheses were included. There were five Ti, five CoCr, and five Y‐TZP frameworks with porcelain veneers (Figure 1). The CoCr and Ti FDPs were made from the same master model. The Y‐TZP FDPs were made from a similar situation using another master model but using the same tooth setup. All the master models had six implants placed at roughly the same position, regio 15,12, 23,25 and between 21/22. However, in the Ti and CoCr models, a fixture was placed in regio 14 while the Y‐TZP had a fixture placed in regio 13. The FDPs were produced on abutment level. The frameworks were used in earlier publications regarding fit (Svanborg et al., 2015, 2019), explaining the difference in implant positioning.

### 2.2 Fabrication of zirconia frameworks

Five stone models (Shera Hard Rock; Shera Werkstoff Technologie GmbH & Co., Lenförde, Germany) of an edentulous maxilla were fitted with six implant abutment replicas (Multi‐unit abutment replica; Nobel Biocare AB, Gothenburg, Sweden). From a tooth setup, an acrylic resin pattern with a cut back design for porcelain veneering was fabricated. The models and the resin pattern were scanned in a laboratory scanner (Nobel Procera, Optimet; Nobel Biocare AB, Gothenburg, Sweden), and designed using CAD software (Nobel Procera Crown & Bridge 4.0.10.5; Nobel Biocare AB, Gothenburg, Sweden). The CAD files were sent to a production center for the manufacturing of Y‐TZP frameworks (Procera; Nobel Biocare AB, Gothenburg, Sweden). The material composition was in accordance with ISO 13356.

### 2.3 Fabrication of CoCr and Ti frameworks

Five models of an edentulous maxilla in type IV stone (Shera Hard Rock; Shera Werkstoff Technologie GmbH & Co., Lenförde, Germany) were fitted with six implant abutment replicas (Ankylos, Balance Base abutment narrow; Dentsply Sirona Implants, Mölndal, Sweden). One acrylic resin pattern simulating a patient case (designed for porcelain veneering) was made from a tooth‐setup. The models and the resin pattern were sent to a production center (Atlantis suprastructures; Dentsply Sirona Implants, Hasselt, Belgium) and for each model two frameworks were fabricated.

The models were scanned with the resin pattern and one framework for each model was computer numerical control (CNC)‐milled in CoCr (Starloy Soft; Dentsply‐DeguDent, Hanau‐Wolfgang, Germany) and one framework CNC‐milled in Ti (Commercially pure, Grade 4). Resulting in five CNC‐milled CoCr frameworks and five CNC‐milled Ti frameworks. For material composition, see Table 1.

**Table 1:** Material composition for CoCr and Ti frameworks (in wt%)

Material	Ti	Al	V	Fe	O	H	C	N	Co	Cr	Mo	W	Si	Mn	Nb
CNC CoCr^a^	‐	‐	‐	7.5	‐	‐	‐	‐	54.1	20.0	‐	16.4	1.5	0.3	0.2
CNC Ti^b^	Bal^c^	‐	‐	>0.50	>0.40	>0.10	>0.10	>0.05	‐	‐	‐	‐	‐	‐	‐

^a^ Starloy Soft (Dentsply‐DeguDent).

^b^ Grade 4.

^c^ Bal = Balance, up to 100%.

### 2.4 Porcelain veneering of zirconia frameworks

The zirconia frameworks were sent to a commercial dental laboratory, and an experienced dental technician performed the porcelain veneering. The procedures, firing cycles, and materials are presented in Table 2. After the veneering procedure, the mating surfaces on the frameworks were air‐abraded with 50 µm glass beads at 2 bar (Magma 50 µm; M‐Tec Dental AB, Malmö, Sweden).

**Table 2:** Porcelain veneering of the zirconia frameworks

*n*	Program	Material	End temp (°C)
1	Liner	IPS e.max Ceram ZirLiner	960
2	Dentin/Incisal	IPS e.max Ceram Dentin/Incisal	730
1	Glaze	IPS Ivocolor Glaze paste	710

*Note*: Firing cycles and materials. Furnace Ivoclar Vivadent Programat P510.

Abbreviation: *n*, number of firing cycles.

### 2.5 Porcelain veneering of CoCr and Ti frameworks

The porcelain veneering of the frameworks was performed at a commercial dental laboratory by experienced dental technicians, according to the manufacturer's instructions. For the CoCr frameworks, GC Initial MC was used and for the Ti frameworks, GC Initial Ti (GC Nordic AB, Älvsjö, Sweden) was used. After veneering, the visible metal surfaces were polished and the mating surfaces on the frameworks were air‐abraded to remove the oxide that had accumulated during the firing cycles with 50 µm glass beads at 2–3 bar (Magma 50 µm; M‐Tec Dental AB, Malmö, Sweden). The procedures, firing cycles, and materials are presented in Tables 3 and 4.

**Table 3:** Porcelain veneering procedure of the CNC‐milled CoCr frameworks

*n*	Program	Material^a^	End temp (°C)
1	Oxidation	N/A	950
1	Bonding	GC Initial Metal bond	980
1	Opaque 1	GC Initial MC Paste Opaque	960
1	Opaque 2	GC Initial MC Paste Opaque	950
2	Dentin	GC Initial MC Dentin	905
		GC Initial MC Enamel	
1	Glaze	N/A	870

*Note*: Firing cycles and materials. Furnace Ivoclar Programat P90.

Abbreviation: *n*, number of firing cycles.

^a^ GC Initial MC, GC Nordic AB, Älvsjö, Sweden.

**Table 4:** Porcelain veneering procedure of the CNC‐milled Ti frameworks

*n*	Program	Material^a^	End temp
1	Bonding	GC Initial Ti Bonder	820°C
1	Opaque 1	GC Initial Ti Powder Opaque	820°C
1	Opaque 2	GC Initial Ti Powder Opaque	820°C
2	Dentin	GC Initial Ti Dentin	820°C
		GC Initial Ti Enamel	815°C
1	Glaze	N/A	795°C

*Note*: Firing Cycles and Materials. Furnace Dentsply DeTrey Multimat C.

Abbreviation: *n*, number of firing cycles; Ti, titanium.

^a^ GC Initial Ti, GC Nordic AB, Älvsjö, Sweden.

### 2.6 Specimen preparation

All FDPs were subjected to thermocycling for 5000 cycles in a thermocycling device (Huber Thermocycler; Peter Huber Kältemaschinenbau AG, Offenburg, Switzerland). The cycling involved two baths of distilled water, the FDPs were placed in a 5°C bath for 30 s, then in another bath of 55°C for 30 s with a transfer time of 5 s between the baths. The thermocycling was performed to resemble the exposure to the environment of the oral cavity (Øilo et al., 2013; Palmer et al., 1992). After the thermocycling, the FDPs were stored in distilled water at room temperature until the loading procedure.

Epoxy models (EpoFix kit; Struers ApS, Ballerup, Denmark) were made with abutment replicas embedded in epoxy. A total of three epoxy models were made, one for each type of FDP material.

### 2.7 Mechanical loading procedure

All tests were performed by the same two investigators, one visually supervising the test, to stop manually if any fracture occurred that the testing equipment did not register. The other supervised the loading graph, to register any drop of load, which was interpreted as an indication of fracture.

**Figure 2:**
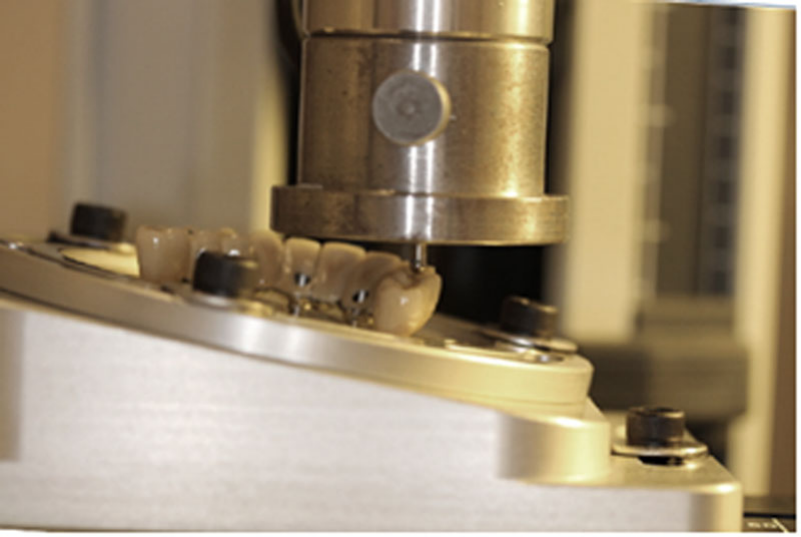
Photograph of the mechanical strength test. Application of the force with a round steel ball in the distal fossa of the occlusal area of the pontic, replacing tooth 24

**Figure 3:**
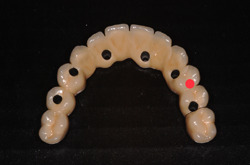
Area of the loading procedure. Test performed in the distal fossa on the pontic replacing tooth 24

The FDPs were fixated, one at a time, on the epoxy model with bridge screws tightened at 15 Ncm and then mounted in a mechanical testing machine (Lloyd LRX, Lloyd Instruments Ltd., Bognor Regis, Great Britain) in a customized jig for angulation. The load was applied with a round steel ball, 3 mm in diameter, with a crosshead speed of 0.5 mm/min. The load was applied with a 10° angle to the longitudinal axis of the specimen, as shown in Figure 2. It was applied in the distal fossa of the occlusal area of the pontic, replacing tooth 24 (Figure 3), until fracture of the veneer was detected either visibly, by acoustic event or drop of load, detected by the Lloyd, which ever came first (Larsson et al., 2012; Nordahl et al., 2015).

Data on loading force, in Newtons, was collected by a computer, processing the data with a software program (Nexygen, Ondio 4,1; Lloyd Instruments Ltd., Bognor Regis, Great Britain). After the loading procedure, FDPs were removed for further analyses.

### 2.8 Qualitative inspection

A microscopic assessment was performed (Nikon Digital Sight DS‐Fi1, Nikon Stereo microscopes SMZ800; Nikon Corporation, Konan, Minato‐ku, Tokyo, Japan) by the same two investigators conducting the mechanical loading procedure. The FDPs were categorized according to qualitative results such as the location of the fracture. It was also evaluated whether the fractures were adhesive, cohesive, or inconclusive. Those named inconclusive were not possible to determine.

### 2.9 Data processing/statistical analysis

Statistical analysis was performed using SPSS Statistics (IBM Corp. Version 25, Armonk, NY, USA). Kruskal–Wallis test was performed to analyze fracture loads to compare porcelain veneer strength. The statistical significance was set at *p* < .05.

## 3 RESULTS

### 3.1 Fracture load

The highest mean fracture load was observed for Ti 1002.8 N and the lowest for CoCr at 775.2 N. The CoCr showed the highest load range (340–1484 N) and the Y‐TZP the lowest (495–1326 N). The fracture loads are presented in Table 5. The Kruskal–Wallis test showed no significant difference between the groups.

**Table 5:** Mean load at fracture (N)

	*n*	Mean load (range) (*N*)	SD	*p* Value
CoCr	5	775.2 (340–1484)	426.75661	NS
Ti	5	1002.8 (387–1467)	388.0782	NS
Y‐TZP	5	854.4 (495–1326)	344.912	NS

Abbreviations: CoCr, cobalt–chromium; Ti, titanium; Y‐TZP, yttria‐stabilized zirconia.

### 3.2 Fracture quality assessment

**Figure 4:**
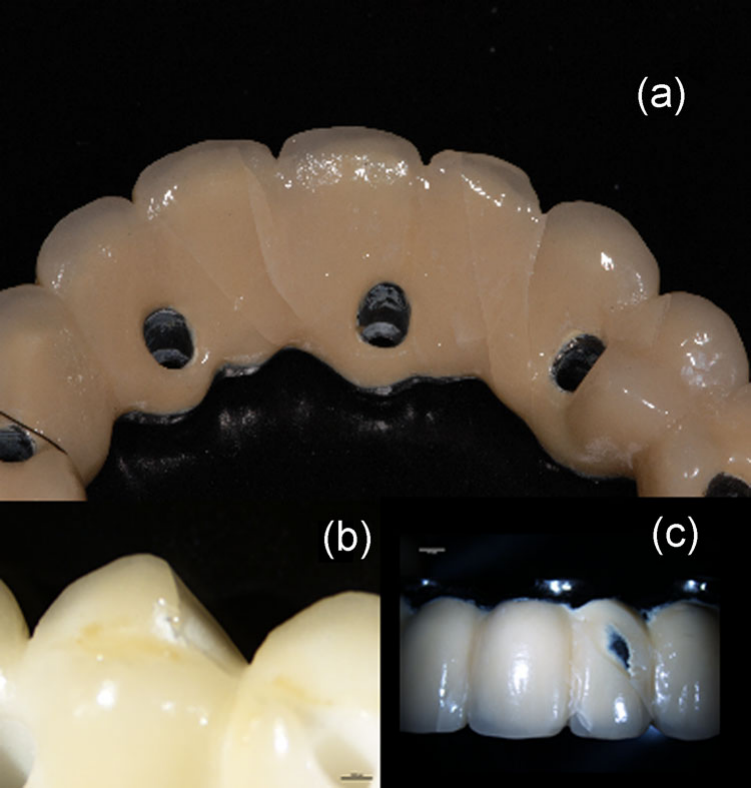
Photographs of veneer fracture appearances (a–c). (a) Ti FDP nr 5 after load showing common appearance with cracks in the 21,22 area rather than chip off in the 24 area. (b) Y‐TZP nr 5 after load. Common/typical appearance of fracture in Y‐TZP FDPs. (c) Buccal view of Ti FDP nr 5 after load showing common appearance with chip off at 22 buccally rather than fracture in the 24 area

**Figure 5:**
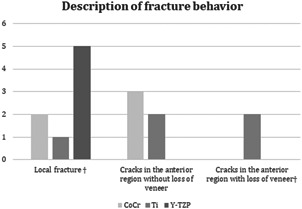
Bar chart, fracture quality assessment. ^†^Depicted in Figure 4b,c

Three different fracture patterns were observed: local fracture, cracks in the anterior region without loss of the veneer, or cracks in the anterior region with loss of veneer. Local fractures were generally placed buccally on 24, while crack formations in the anterior region were running lingually or buccally as depicted in Figure 4. The Y‐TZP FDPs showed only local fractures in the loaded area while CoCr and Ti more often exhibited cracks in the anterior region. For Ti FDPs, loss of veneer was observed in two cases (Figure 5).

**Figure 6:**
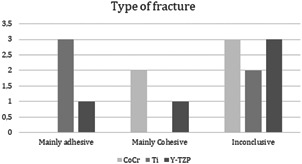
Bar chart results based on fracture type

In many fractures, it was not possible to determine whether the fracture was adhesive or cohesive. None of the CoCr FDP was considered clear adhesive or cohesive fracture while three of the Ti FDPs were considered adhesive (Figure 6).

## 4 DISCUSSION

The results from this study demonstrated no statistically significant difference with respect to porcelain veneer fracture strength in maxillary full‐arch bridges made of Ti, CoCr, or Y‐TZP. However, there was a large heterogeneity within each material group affecting the outcome. The appearances of the fractures differed between the groups, indicating fracture behavior differences. The null hypothesis was therefore only in parts accepted.

Several studies have reported that the average bite forces range from 300 to 800 N in the posterior region (Helkimo et al., 1977; Koc et al., 2010). However, implant‐supported FDPs are known to be exposed to larger forces than natural teeth (Jacobs & van Steenberghe, 1993) which may be a result of lesser tactile sensibility (Higaki et al., 2014). In this study, the forces applied varied between 340 and 1484 N indicating that some of the FDPs failed within the range of clinically relevant forces.

No power calculation was performed since the number of FDPs was set to the available number of FDPs of each framework material. A larger number of samples might have resulted in a more pronounced and perhaps statistically significant difference.

A pilot test was performed using one CoCr FDP before the present study was conducted. In this pilot, a plastic foil was used between the steel ball and the FDP to avoid contact damage and distribute forces evenly (Kelly, 1999; Øilo et al., 2013). This was later excluded, after a recommendation from a Lloyd supplier, due to problems with the steel ball sliding on the FDP during loading and resulting in difficulties keeping the intended direction of the load. To assure a standardized loading protocol, a different steel ball with the same measurements was used but without the plastic foil. The pressure of the ball to the veneer surface may create a defect within the porcelain. Contact points may therefore have been affected, causing contact damage and crack formation in a way that is not comparable with a clinical situation (Øilo et al., 2013). Moreover, the steel ball has different properties compared to the opposing teeth and therefore the actual load should not be translated directly to the clinical situations. The load was applied in angulation of 10° in accordance with other similar in vitro studies to simulate the natural direction of load as the opposing dentition (Larsson et al., 2007, 2012; Löfgren et al., 2017; Nordahl et al., 2015). Nordahl et al. has described that angulation creates more complex stress by adding elements of tensile forces rather than pure compressive forces (Larsson et al., 2012). In this study, a 3 mm diameter ball was used. According to Kelly et al. the optimal diameter should be at least 40 mm to develop representative tooth‐to‐tooth contacts (Kelly, 1999). However, it is common to use a smaller indenter, as seen in numerous in vitro studies and the loading conditions were similar for all test subjects justifying the comparison (Larsson et al., 2007, 2012; Nordahl et al., 2015; Quinn et al., 2010).

The computer was set to stop automatically if load dropped quickly but, after the pilot test, a manual stop was considered more sensitive. Therefore, manual supervision and halt were added. In contrast to other studies, there were difficulties in detecting an audible crack in the veneer (Bakitian et al., 2019). However, care was taken to register the first sound, visible crack, or loading drop.

The FDP frameworks in this study were designed in CAD programs and milled. Each framework was designed to be optimal for the specific material and the design of the different framework groups was not identical. Furthermore, the veneering was made by hand. Therefore, it would be reasonable to assume that there are small differences in the veneering designs, that is, thickness, shape and polishing, as well as internal defects in the veneer (Kelly et al., 1989). The impact of the latter on the result remains to be questioned. However, this is the reality in a clinical situation and thus relevant for the outline of the study. The design of the FDPs may also affect the load‐bearing capacity (Bakitian et al., 2019; Larsson et al., 2007). Usually, when comparing different materials or designs, in vitro studies are based on the same models where teeth or implant placement are identical (Bakitian et al., 2019; Karl et al., 2007; Larsson et al., 2007; Löfgren et al., 2017). In this study, the models were identical within the metal framework groups. The Y‐TZP FDPs were, however, made from a different master model not completely identical with the metal framework groups, which may play an unclear role in the results. The selected location of load, distal fossa of 24, was with consideration to the screw access hole and implant placement to make the test situation as comparable as possible. The pontic of 24 had implant support directly on each side in all three FDP groups and no screw access hole, as shown in Figure 3. The FDPs are all produced on the abutment level, reducing a difference in the implant system design. The screw access hole may have a role to play in the frequency of fractures of the veneer as shown, in both in vitro studies and reviews (Karl et al., 2007; Wittneben et al., 2014). In the present study, however, it should not play a significant role in the comparison between frameworks since all the FDPs were screw‐retained and the area of loading was not in contact with any access holes.

Previous results have shown that if there is a complete fracture of the Y‐TZP framework, it usually happens in the weakest point of the FDP, often the connector (Bakitian et al., 2019; Le et al., 2015). None of the FDP in this study was loaded to complete fracture but the fracture of the veneer occurred more often in the area of loading. However, the Ti FDPs showed more fractures and cracks in the anterior region than CoCr and Y‐TZP FDPs. One reason for this, may be the different E‐modulus of the different framework materials, CoCr (200 GPa according to manufacturer) and Y‐TZP (210 GPa) have about twice the E‐modulus compared to Ti (105 GPa). Since CoCr and Y‐TZP have quite similar E‐modulus and yet differ in fracture appearances, it cannot be the only explanation (Anusavice et al., 2013). However, the results need to be carefully interpreted as there was a difference in master model design, which might have had an influence on the outcome. Also, there was a small number of study samples and further large scale studies need to be made to confirm these results.

The results from the present study demonstrated no statistically significant difference in mean fracture load within the test groups but other studies have shown that veneering materials for Y‐TZP have a lower fracture strength than veneering materials used for porcelain fused to metal (Quinn et al., 2010). It was not shown whether adhesive or cohesive fractures were more common. Choi et al. (2009) reported that metal‐porcelain, when studying rectangular specimens, has higher shear bond strength than zirconia‐porcelain, and that fractures appeared to be more cohesive for zirconia (Choi et al., 2009). The results from the present study are not possible to apply directly to a situation where the specimens are shaped like teeth. The frameworks in the present study were designed to be ideal for each type of framework material and were not identical in dimensions and shapes in between the material groups. In addition, it is probably a larger variation in shape and thickness of veneering porcelain for tooth shaped test specimens due to differences in framework design and veneering procedure compared to a rectangular shape with a straight surface and a uniform thickness.

## 5 CONCLUSIONS

Within the limitations of this study, the conclusion was that framework material may affect the fracture behavior of maxillary full‐arch bridges; however, there were no differences in veneer fracture strength when frameworks of Ti, CoCr, or Y‐TZP were compared.

## AUTHOR CONTRIBUTIONS

**Märta Vahnström**: First author, conceptualization, methodology, investigation, analysis, writing the original draft. **Petra Hammarström Johansson**: Conceptualization, methodology, investigation. **Per Svanborg**: Corresponding author, conceptualization, supervision. **Victoria Franke Stenport**: Conceptualization, methodology, analysis, supervision.

## CONFLICTS OF INTEREST

The authors declare no conflicts of interest.

## Data Availability

The data that support the findings of this study are available from the corresponding author, [MV], upon reasonable request.
